# Advances in Plant-Derived Extracellular Vesicles: Implications for Apple-Derived EVs

**DOI:** 10.3390/plants14223425

**Published:** 2025-11-09

**Authors:** Hao Fu, Shunyuan Yong, Yanping Song, Jiangbo Dang, Danlong Jing, Di Wu, Qigao Guo

**Affiliations:** 1 Key Laboratory of Agricultural Biosafety and Green Production of Upper Yangtze River (Ministry of Education), Chongqing Key Laboratory of Forest Ecological Restoration and Utilization in the Three Gorges Reservoir Area, College of Horticulture and Landscape Architecture, Southwest University, Beibei, Chongqing 400715, China; fhplants@163.com (H.F.); yongshunyuan2022@163.com (S.Y.); w963590198@163.com (Y.S.); dangjiangbo@126.com (J.D.); jingdanlong@swu.edu.cn (D.J.); wudisuper@swu.edu.cn (D.W.); 2 Academy of Agricultural Sciences, College of Horticulture and Landscape Architecture, Southwest University, Chongqing 400715, China; 3 Department of Applied Biological Chemistry, Graduate School of Agricultural and Life Sciences, The University of Tokyo, 1-1-1 Yayoi, Bunkyo-ku, Tokyo 113-8657, Japan

**Keywords:** plant-derived extracellular vesicles (PDEVs), apple-derived extracellular vesicles (ADEVs), exosome-like extracellular vesicles, biological activity, drug delivery

## Abstract

Plant-derived extracellular vesicles (PDEVs) are nanoscale membrane vesicles released by edible plants that deliver proteins, lipids, nucleic acids, and small metabolites to recipient cells, thereby modulating inflammation, barrier function, metabolism, and intercellular signaling. In recent years, PDEV research has advanced from concept and in vitro observations to engineering-ready systems with validation in animal models, encompassing oral, transdermal, and intranasal delivery paradigms. Among edible plants, the apple has broad consumption and a favorable safety profile; however, studies on apple-derived extracellular vesicles (ADEVs) lag behind those on other plant EVs. Accordingly, this review systematically summarizes ADEV progress across extraction methods, characterization, molecular cargo, and roles in disease settings. We highlight evidence gaps in animal efficacy and translation, and propose priorities including process standardization, harmonized critical quality attributes, in vivo biodistribution, and long-term safety. Our aim is to provide a reference for ADEV research and to accelerate the development of safe, low-cost, scalable bionanocarriers for disease therapy.

## 1. Introduction

**Figure 1:**
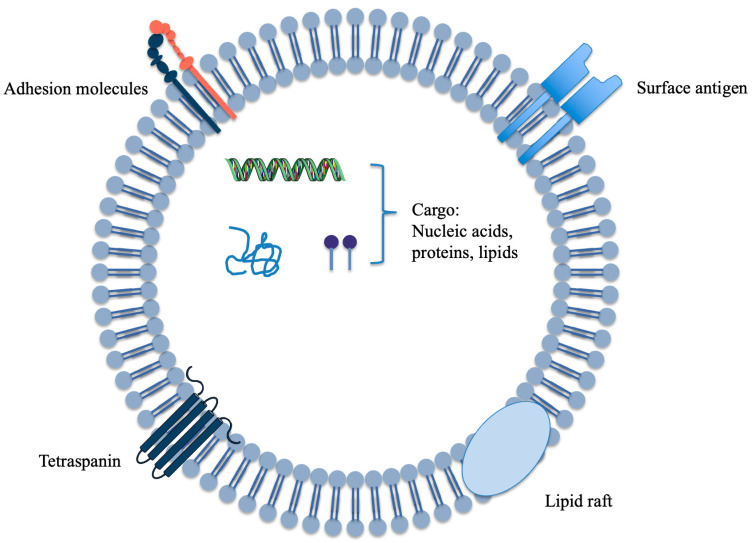
Schematic of an extracellular vesicle from plant. In this review, we synthesize the latest advances in plant EV research and, drawing on current findings for ADEVs, assess their biological functions and drug-delivery potential. We compare EVs from different plant sources to delineate commonalities and distinctions, and we outline prospective applications in disease prevention, therapy, and delivery. Our aim is to provide a conceptual reference and developmental roadmap to inform future in-depth studies and clinical translation.

Extracellular vesicles (EVs; ~30–150 nm in diameter) are released upon fusion of multivesicular bodies with the plasma membrane and carry bioactive cargo—including proteins, nucleic acids, and lipids—thereby mediating intercellular signaling and modulating both homeostasis and disease progression (Figure 1) [1,2]. Historically, research has focused on animal-derived EVs (A-EVs), yet translational barriers regarding source traceability, batch-to-batch quality consistency, manufacturing cost, and immunological safety remain unresolved. In response, plant-derived extracellular vesicles (PDEVs) have attracted increasing attention owing to their broad availability, edibility, low immunogenicity, and favorable biocompatibility. Plant EVs were first reported in 2008 and have since been identified across multiple edible species, demonstrating potential in inflammation regulation, maintenance of gut homeostasis, anti-tumor activity, and drug delivery [3,4,5]. Apples (*Malus domestica*), as one of the most widely cultivated and consumed fruit trees worldwide, provide an ideal model for studying PDEVs. This species benefits from well-established genomic resources, standardized cultivars, and extensive biochemical and metabolomic datasets [6]. Despite a relatively late start, apple-derived extracellular vesicles (ADEVs) have achieved substantive progress: protocols for isolation and characterization are becoming increasingly standardized; preliminary functional signals have emerged regarding epithelial homeostasis, mucosal immunity, and skin-barrier protection; and initial human-facing data on safety and usability have appeared, together providing foundational evidence for potential nutritional interventions and drug-delivery applications [7,8,9]. In recent years, PDEVs have been widely anticipated owing to their safety profile and scalability, and have even been proposed as candidates for novel functional foods or oral drug-delivery vehicles [10].

## 2. Search Strategy (Databases, Search Terms, and Criteria)

This review was conducted based on a systematic search of the literature to identify studies relevant to the isolation, characterization, mechanism of action, and therapeutic applications of plant-derived extracellular vesicles (PDEVs), with a specific focus on apple-derived extracellular vesicles (ADEVs). The literature search was performed up to October 2025 using the primary databases PubMed, Web of Science, and Scopus.

The search strategy involved combining primary keywords related to the nanocarriers: (Plant-derived extracellular vesicles OR plant exosomes OR exosome-like nanovesicles) AND (ADEVs OR apple-derived OR fruit-derived); with terms related to their application and biology: (Therapy OR delivery OR cancer OR inflammation OR metabolism); Specific examples of search strings included: “plant exosomes AND apple”, “ADEVs therapy”, and “plant-derived extracellular vesicles delivery”.

To ensure the rigor and relevance of the selected literature, we applied specific Inclusion and Exclusion Criteria. The inclusion criteria focused on original research articles and comprehensive reviews published in English. Conversely, we excluded non-peer-reviewed materials such as conference abstracts, book chapters, preprints without peer review, and dissertations, along with articles not published in English or those focused exclusively on synthetic nanoparticles.

## 3. Isolation and Characterization

**Figure 2:**
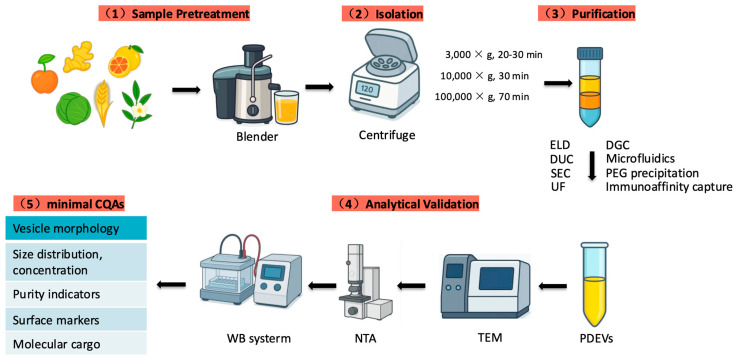
Standardized workflow for the isolation and characterization of plant-derived extracellular vesicles (PDEVs). Most existing studies on ADEVs follow these standardized strategies. Although the overall methodology remains in an early stage, a reliable workflow for isolation and characterization has begun to emerge, providing a foundation for subsequent functional validation and translational development (Table 1).

The isolation and characterization of extracellular vesicles (EVs) are essential prerequisites for elucidating their molecular composition and biological functions, and they are critical steps toward applications in medicine and food science. In this section, we summarize current extraction methods for plant-derived extracellular vesicles (PDEVs) and compare methodological differences to guide the optimization of the preparation workflow for ADEVs. The aim is to enhance both purity and yield while preserving vesicle integrity and bioactivity. As illustrated in (Figure 2), the standardized workflow for PDEVs’ isolation and characterization consists of five major steps: sample pretreatment; isolation by differential centrifugation; purification; analytical validation, and evaluation of minimal critical quality attributes (CQAs).

**Table 1:** Isolation and Characterization Workflows for Plant-Derived Extracellular Vesicles, with Current Status in Apple-Derived EVs.

Method	Principle	Advantages	Limitations	Applications in PDEVs	Reference (ex.)	Current Status in ADEVs
Differential ultracentrifugation (DUC)	Stepwise centrifugation to remove debris and organelles, then high g-force to pellet EVs.	Widely available; reproducible starting workflow; scalable sample volumes.	Lower purity vs. DGC/SEC; co-precipitation of soluble proteins/small molecules; time-consuming.	Grapefruit juice; Ginger; *Arabidopsis* leaves.	[11,12,13]	ADEVs: primary workflow; combine with density-gradient centrifugation when needed.
Density-gradient centrifugation (DGC)	Separation by buoyant density in sucrose/iodixanol gradients to resolve EV subpopulations.	High purity; resolves subfractions; reduces contaminants.	Labor-intensive; long runtime; requires ultracentrifuge and gradients.	Grapefruit; Almond; *Panax notoginseng*.	[14,15,16]	Occasionally used as a polishing step after DUC; not routine in apples.
Size-exclusion chromatography (SEC)	Fractionation by size on a porous matrix; EVs elute in early fractions.	Higher purity; reduces soluble protein/small-molecule carryover; preserves integrity.	Requires dedicated columns/instrumentation; lower throughput; fraction handling needed.	Carrot; Tomato; Blueberry.	[17,18,19]	UC followed by SEC has been reported for apples; broader, systematic SEC-only validation remains limited.
PEG precipitation	Polyethylene glycol competes for water, reducing EV solubility and precipitating vesicles.	Simple; inexpensive; relatively high yield; amenable to scale.	Co-precipitation of proteins/impurities; purity limited—prefer as pre-concentration.	Ginger (PEG6000).	[20]	ADEVs: rarely used; if applied, follow with SEC to remove contaminants.
Ultrafiltration (UF)	Size-based retention through membranes with defined MWCO for concentration/rough fractionation.	Rapid; low cost; compatible with serial processing; no special rotors.	Membrane clogging; shear stress risk; alone may affect integrity/purity.	Commonly used as pre-concentration, then coupled with SEC for purification.	[20]	Rarely used; if applied, follow with SEC to remove contaminants.
Immunoaffinity capture	Antibody–antigen recognition of EV surface markers (e.g., tetraspanins) for selective capture.	High specificity and purity; enables subpopulation isolation.	Depends on known markers; cost; potential yield trade-offs.	*Arabidopsis* TET8^+^ EVs.	[21]	ADEVs: lack of universal surface markers—routine application not established.
Microfluidics	On-chip fractionation by size, charge, or affinity using engineered microstructures/fields.	High-throughput potential; small sample requirement; precise control.	Specialized chips; standardization and scalability still developing.	Exploratory in PDEVs (method development stage).	[22,23]	ADEVs: no mature application reported.
Electrophoresis–dialysis (ELD)	Electric field–assisted migration with concurrent dialysis to separate EVs from small solutes.	No large equipment; feasible in standard labs; yields comparable to UC in some studies.	Cross-source reproducibility and scale-up to be validated.	Lemon (ELD).	[24]	ADEVs: not yet systematically validated.

### 3.1. Isolation and Purification Methods

The mainstream workflow for obtaining PDEVs centers on differential ultracentrifugation, optionally coupled with density-gradient separation to enhance purity and homogeneity. This combination is widely regarded as a generalizable and reproducible starting protocol. Size-exclusion chromatography (SEC) can further reduce co-isolation of soluble proteins and small molecules across diverse sources, yielding particle fractions that are better suited for functional assays; when used in tandem with ultracentrifugation, SEC improves sample quality without substantially compromising yield [12,18,25]. Polyethylene glycol (PEG) precipitation and ultrafiltration are useful as auxiliary steps for pre-concentration or rapid screening; however, when deployed as stand-alone methods, limited purity and contaminant co-precipitation can constrain the interpretability and cross-study comparability of mechanistic and in vivo investigations [26]. Immunoaffinity capture and microfluidic platforms offer prospects for high specificity and throughput, but in plant systems broader adoption remains limited by the scarcity of universal markers and cost considerations; consequently, these approaches largely remain at the methodological exploration stage [21,22].

For ADEVs specifically, most studies employ a baseline protocol built around differential ultracentrifugation, with density-gradient ultracentrifugation added as needed. This framework yields morphologically and size-consistent vesicle populations that support downstream functional validation. Systematic head-to-head comparisons with SEC remain limited; multi-laboratory evaluations under a unified characterization checklist are therefore warranted to standardize and optimize ADEVs isolation while preserving vesicle integrity and bioactivity.

Beyond method choice, the agricultural origin of plant materials materially affects both yield and safety. In plants, extracellular vesicles can participate in the sequestration and transport of xenobiotics, including pesticides and microbicides. Thus, vesicles isolated from intensively farmed fruits and vegetables may co-concentrate chemical residues, potentially compromising the safety of products intended for food, cosmetic, or delivery applications. Conversely, comparative studies have indicated that organically cultivated fruits and vegetables yield higher quantities of PDEVs with enhanced antioxidant activity and biofunctionality compared to conventionally farmed counterparts [10,27,28]. Accordingly, future studies and industrial pipelines should prioritize controlled, pesticide-free cultivation systems and implement rigorous residue testing during PDEVs isolation to ensure functional efficacy and consumer safety.

From a manufacturing perspective, while differential ultracentrifugation remains the laboratory workhorse, its low throughput, high energy demand, and poor linearity upon scale-up limit suitability for large-batch production [29]. Traditional approaches based on plant cell or callus cultures are likewise difficult to scale and raise regulatory concerns due to the use of exogenous growth factors and antibiotics, which must be completely removed to ensure safety and compliance [29]. Therefore, large-scale, food-compatible production should rely on direct extraction from edible plant tissues and agro-industrial streams (e.g., juice, puree, pomace, peels) rather than cell-culture–derived systems [30].

In practice, a scalable and industry-aligned route—particularly for ADEVs—is to integrate tangential-flow filtration (TFF) for bulk concentration and diafiltration with membrane chromatography as a polishing step to reduce soluble proteins, DNA, and process-related impurities [30]. SEC remains valuable for analytical or pilot-scale fractionation, whereas PEG precipitation and stand-alone ultrafiltration are best reserved for pre-concentration/screening due to purity trade-offs when used as primary capture [31]. Finally, validated stabilization (e.g., lyophilization or low-temperature spray-drying with disaccharide protectants) supports batch-to-batch quality consistency while preserving vesicle integrity and bioactivity, providing a realistic pathway toward regulatory-ready, scalable PDEVs preparations [32].

Taken together, a coherent strategy emerges: adopting reproducible laboratory workflows for discovery, source-control and residue-testing for safety, and TFF-centered downstream processing for scale-up, with appropriate polishing and stabilization steps to ensure product quality and functionality.

### 3.2. Characterization and Quality Control

Baseline morphological and physicochemical characterization primarily relies on transmission electron microscopy (TEM) and nanoparticle tracking analysis (NTA): the former confirms canonical vesicular morphology, while the latter reports size distributions and particle concentrations; cryo-electron microscopy can be incorporated, when needed, to minimize preparation-induced artifacts [33,34]. At the molecular level, a minimal characterization panel should encompass proteins, lipids, and small RNAs. Western blotting or mass spectrometry is used to verify representative molecules associated with vesicle biogenesis, membrane trafficking, or stress responses; lipidomics confirms major constituents such as glycerophospholipids and sphingolipids and monitors inter-batch variation; and small-RNA sequencing defines the miRNA cargo repertoire, providing candidates to support cross-kingdom regulatory hypotheses [25,35]. Unlike mammalian EVs, plant systems currently lack unified, cross-species membrane markers; consequently, most studies adopt a composite framework that integrates morphological, physicochemical, and multi-omics evidence to ensure source attribution and functional comparability [21].

In the context of ADEVs, TEM and NTA have yielded broadly consistent readouts of morphology and size. At the omics level, reproducible protein and small-RNA cargo profiles have also been reported, indicating that prevailing isolation workflows can produce source-stable and comparable vesicle populations. Nevertheless, harmonized critical quality attributes (CQAs)—for example, batch-to-batch consistency criteria and thresholds for residual soluble proteins and small molecules—together with multi-center verification, remain to be established to strengthen comparability across experiments and preparation batches [7,8,9].

## 4. Molecular Composition and Biological Functions

The biological effects of PDEVs are tightly linked to their cargo—proteins, lipids, nucleic acids, and secondary metabolites. Species identity, tissue of origin, and isolation workflow can all introduce substantial differences in cargo composition, relative abundance, and molecular configuration; these differences, in turn, affect vesicle stability, cellular uptake routes, in vivo distribution, and the magnitude of pathway modulation [36,37].

### 4.1. Proteome

Proteins are integral components of extracellular vesicles (EVs): they maintain vesicular architecture and directly mediate intercellular communication. Current evidence indicates that the protein repertoire of plant EVs is not a simple mirror of the soluble proteome of the source tissue; rather, it exhibits selective enrichment [38]. Common categories include cytoskeletal and cytosolic proteins, factors involved in membrane transport and vesicle biogenesis, proteins related to stress responses and defense, and lineage-specific proteins in certain taxa [39,40]. Reproducible qualitative and quantitative differences have been observed across plant sources and isolation methods. For example, citrus-derived samples more frequently feature heat-shock proteins (e.g., HSP70, HSP80) and coat/trafficking factors (e.g., clathrin heavy chain) [41,42], whereas members of the Apiaceae and Liliaceae families often show enrichment of coat proteins and chaperones associated with budding, endocytosis, and vesicular transport [43]. Although TET family four-pass transmembrane proteins, HSP70, and aquaporins have been recurrently proposed as candidate markers, a universally accepted, cross-species marker set for plant EVs is still lacking. The positive marker lists reported across studies remain strongly influenced by methodological variables, including sample origin, buffer systems, and centrifugation or chromatographic workflows [44].

In the limited literature on ADEVs, Trentini [45] identified 187 Malus domestica proteins in extracellular vesicles isolated from the ‘Golden Delicious’ cultivar using LC–MS/MS, representing the most systematic characterization of the ADEVs proteome to date. These proteins predominantly include metabolic enzymes, membrane transporters, and stress-response factors, suggesting potential roles for ADEVs in cargo transport, metabolic regulation, and cellular protection.

### 4.2. Lipidome

Lipids are core constituents of the bilayer in PDEVs. They determine vesicle structural stability and interactions with recipient cells, and they participate in transmembrane transport, immune modulation, and tissue distribution [10]. Plant EVs typically feature glycerophospholipids as the predominant class, with variable proportions of glycolipids and sphingolipids. Common subclasses include phosphatidylethanolamine (PE) and phosphatidylcholine (PC), with detectable phosphatidylserine (PS) and minor phosphatidylinositol (PI); in some sources, plant-specific galactolipids—digalactosyldiacylglycerol (DGDG) and monogalactosyldiacylglycerol (MGDG)—are also abundant [38].

Quantitatively, these subclasses differ markedly among species, yielding distinguishable lipid signatures. Vesicles from Rutaceae (e.g., citrus, grapefruit) often display higher proportions of PE and PC [46], whereas rhizome-derived vesicles such as those from ginger repeatedly show enrichment of phosphatidic acid (PA) alongside MGDG and DGDG [47]. Such differences relate to membrane curvature, fusogenic capacity, and in vivo stability; in several plant sources, sphingolipids are also elevated and implicated in signaling [48]. In addition, because mammalian macrophage receptors (e.g., the CD300 family) can recognize PE/PS, these membrane-lipid features could influence interactions between plant vesicles and phagocytes, although direct functional validation for specific plant vesicles remains limited [49].

Recent lipidomics data on ADEVs from the ‘Golden Delicious’ cultivar annotated ~158 lipid species, of which >80% were glycerophospholipids. PE constituted the dominant subclass (~40–60%), followed by PC, ceramide (Cer), and PS. Notably, cultivation regime correlated with lipid profiles: conventionally grown samples (GD) exhibited higher relative abundances of diacylglycerols/triacylglycerols (DAG/TAG), Cer, PC, and PE, whereas organically grown samples (GDBio) were enriched in lysophospholipids (LPC, LPE) and PI. These findings suggest that agricultural practices can modulate ADEVs membrane composition; in particular, shifts in lysophospholipid abundance warrant further evaluation for potential links to bioactivity [27].Collectively, these data provide a systematic basis for the ADEVs lipidome and indicate that different production systems may confer distinct functional potentials.

### 4.3. Nucleic Acids

PDEVs encapsulate multiple classes of nucleic acids, including mRNAs, non-coding RNAs (ncRNAs), small RNAs (sRNAs), DNA, and microRNAs (miRNAs) [50]. Among these, miRNAs—~22 nt in length—are most frequently detected and are increasingly recognized as cross-kingdom regulatory agents: by directing target mRNA degradation or translational repression, they modulate gene expression in physiological and pathological processes such as inflammation, oxidative stress, apoptosis, and tumorigenesis [51].

Sequencing analyses across edible plant sources have identified substantial repertoires of miRNA families and members, with reproducible differences in lineage and abundance attributable to species, cultivar, and isolation workflow. For example, blueberry-derived ELNs (B-ELNs) contain ~140 miRNAs [52]; tomato-derived ELNs (SL-ELNs) harbor ~173 distinct miRNAs [34]; and fresh Rehmannia ELN-like nanoparticles (FRELNs) include 12 miRNAs [53].

Research on ADEVs remains at an early stage. To date, 20 miRNA families have been identified in conventional apples, increasing to 25 families in organically cultivated samples [27]. ADEVs and related apple extracellular vesicles have also been employed in assays that report nucleic-acid encapsulation and stability: (i) 3′UTR reporter systems, small-RNA profiling, and RNase-protection experiments confirm the presence of miRNAs and their protection within vesicles [7,9]; and (ii) under acidic conditions simulating gastric fluid, particle integrity and size distributions are maintained, indirectly supporting physicochemical stability of the nucleic-acid cargo [39,54].

### 4.4. Secondary Metabolites

Secondary metabolites constitute another important cargo class in PDEVs and are widely recognized for diverse therapeutic properties. Emerging evidence indicates that many edible-plant PDEVs carry source-specific secondary metabolites, with selective loading rather than indiscriminate carryover. Representative compounds directly detected and confirmed by chromatographic and mass-spectrometric analyses include: multiple gingerols and shogaols (6-/8-/10-gingerol, 6-shogaol) enriched in ginger vesicles—at levels markedly higher than in matched tissue sections [55]; sulforaphane, a glucosinolate-derived isothiocyanate, concentrated at the nanoparticle level in broccoli vesicles [56]; naringin detected in grapefruit vesicles [57]; vitamin C identified in lemon and strawberry vesicles [58]; and polyphenols such as EGCG enriched in Camellia (tea) vesicles [59]; In addition, oats and lemons have yielded vesicles enriched in β-glucans and pectins enriched in galacturonic acid, respectively [41], suggesting that PDEVs can serve as nanoscale carriers for diet-derived small molecules—including certain polysaccharides.

Not all plant bioactives are packaged into vesicles. Comparative analyses of food matrices and their cognate vesicles have shown that certain orange-juice actives—such as vitamin C and naringenin—are not loaded into orange-derived vesicles, indicating a loading process that is selective and dependent on both molecule class and source [40]. Particle class and isolation route also influence detectability: in broccoli systems, sulforaphane is more enriched in “nanoparticles” than in “microparticles,” and is scarcely detectable as a free form in crude extracts; in ginger, vesicles exhibit pronounced enrichment of gingerols relative to matched tissue sections. These observations underscore the need for size-fractionation coupled with quantitative analysis when profiling vesicle-associated metabolites [39].

With respect to ADEVs, direct and quantitative catalogs of small-molecule secondary metabolites remain scarce. Some studies have suggested the presence of flavonoids and furanocoumarins in apple vesicles, but systematic quantification and cross-method verification have yet to be established [54].

## 5. Applications of PDEVs in Disease Therapy

**Figure 3:**
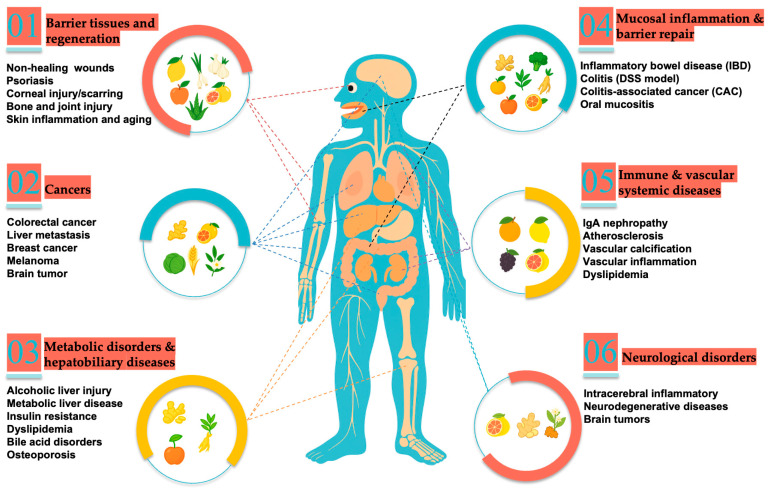
Overview of Plant-Derived extracellular vesicles in Human Diseases.

As research on plant-derived exosome-like nanoparticles (PDEVs) continues to advance, growing evidence demonstrates their remarkable advantages in tumor microenvironment modulation, nucleic acid and small-molecule delivery, oral stability, and biocompatibility, providing new avenues and platforms for interventions against cancer and other major diseases (Figure 3) [60]. To achieve precise delivery and functional applications, researchers have developed multiple molecular loading strategies, including passive incubation, electroporation, sonication, freeze–thaw or extrusion, and saponin-assisted permeabilization [61]; additionally, PEG-based and microfluidic encapsulation methods have been shown to significantly enhance loading efficiency and batch reproducibility, enabling stable and efficient delivery of small molecules and nucleic acids [62]. Current research priorities focus on elucidating mechanisms of action, improving loading efficiency and targeting specificity, achieving industrial-scale production with consistent quality between batches, and systematically evaluating in vivo safety as well as pharmacokinetic and pharmacodynamic characteristics (PK–PD). Overall, these efforts aim to lay a solid foundation for the clinical translation of PDEVs in cancer therapy and other major disease interventions. Studies supporting these efforts span diverse evidence levels, from in vitro and animal models to preliminary human investigations; however, due to the exploratory and overlapping nature of the literature, the current research is often functionally organized (e.g., anti-inflammatory, anti-tumor effects) rather than strictly by evidence level, with the overall goal of laying a solid foundation for the clinical translation of PDEVs in disease therapy.

### 5.1. Barrier Tissues and Regeneration

Focusing on barrier tissues such as skin, the ocular surface, and bone and cartilage, research on PDEVs has progressed from purely in-vitro pro-repair signals to parallel advances in efficacy validation in animal models and integration with materials-based delivery systems. In cutaneous wound repair, lemon-derived extracellular vesicles combined with gelatin methacrylate (GelMA) accelerated closure in diabetic wound models; proposed mechanisms include macrophage phenotype reprogramming, enhanced endothelial and fibroblast activity, and collagen remodeling [63]. In psoriasis models, vesicles derived from scallion and garlic upregulate NRF2 and suppress downstream genes in the IL-17 axis, thereby alleviating cutaneous inflammation. Ex vivo porcine-skin assays further show that both vesicle types reach the suprabasal epidermis and exhibit superior transdermal penetration compared with apple-derived vesicles, suggesting that source-dependent differences and effective depth may influence topical efficacy [64]. Overall, there is growing consensus around a “plant vesicles plus hydrogel” strategy for tissue regeneration, with standardized endpoints recommended for local sustained exposure, pro-angiogenesis, and matrix remodeling [65].

For ocular-surface repair, EVs from plant and non-plant sources accelerate corneal epithelial healing and reduce scarring; consistent with this, aloe-derived small EV-like vesicles exhibit antioxidant and pro-healing activities, supporting their feasibility as active ingredients in eye drops or mucoadhesive formulations, although additional animal and clinical data are needed [66]. In bone and cartilage regeneration, functionalized-scaffold strategies using EVs from diverse sources are relatively mature, and evidence for plant vesicles is accumulating. Studies and reviews indicate that vesicles from grapefruit and other plants may promote wound repair and modulate matrix metabolism, with future potential for intra-articular delivery in fractures and osteoarthritis [67].

Regarding ADEVs, evidence now spans mechanisms through to human application. At the cellular level, ADEVs suppress the TLR4–NF-κB inflammatory pathway, reduce expression of MMP-1, MMP-8, and MMP-9, and increase expression of type I and type III collagen genes—collectively indicating concurrent anti-inflammatory effects, reduced matrix degradation, and enhanced matrix synthesis, with implications for mitigating inflammaging [68]. In a human follow-up study, a 2% ADEVs topical facial formulation evaluated over 60 days, after standardized safety testing, produced significant reductions in erythema (*p* < 0.05) and time-dependent improvements in wrinkle length, total volume, and roughness (wrinkle depth did not reach significance), indicating clinically usable soothing and anti-aging effects [69]. In psoriasis-related work, ADEVs showed pro-differentiation and anti-inflammatory activity in vitro but exhibited weaker transdermal penetration than garlic- and scallion-derived vesicles in ex vivo porcine skin, supporting the rationale for combination formulations or penetration-enhancement strategies [64]. In addition, combining PDEVs from multiple botanical sources may broaden the spectrum of bioactive cargos and complementary activities relative to single-source preparations, thereby enhancing overall biological efficacy.

### 5.2. Cancers

In recent years, research on PDEVs in oncology has progressed from in-vitro activity observations to efficacy validation in animal models and the development of engineering-ready delivery strategies. Two major lines of inquiry have emerged. The first leverages the intrinsic bioactivity of the material—modulating immune responses, suppressing inflammation, and altering stress and metabolic pathways—to inhibit tumor growth and metastasis. The second uses PDEVs as drug-delivery platforms: taking advantage of the low immunogenicity and oral accessibility of vesicles with edible-plant lipid membranes to load chemotherapeutics or nucleic acids for targeted delivery to tumors or inflamed microenvironments, thereby amplifying therapeutic efficacy [37].

For colorectal cancer and colitis-associated cancer, ginger-derived extracellular vesicle–like nanoparticles (ginger-derived ELNs; GELNs) provide some of the most comprehensive evidence. Nanocarriers rebuilt from ginger lipids efficiently load doxorubicin and are taken up by colon cancer cells, achieving superior intratumoral delivery and antitumor effects compared with the free drug (e.g., Colon-26 models), thus exemplifying an engineering strategy that couples edible-source lipids with chemotherapeutics [70]. In addition, grapefruit-derived nanovectors (GNVs) can be functionalized with activated leukocyte-membrane receptors to exploit inflammatory chemotaxis, thereby homing more precisely to inflamed tumor tissue and markedly increasing lesion accumulation and tumor inhibition; the same approach has been used to deliver miR-18a, which induces hepatic macrophage M1 polarization and suppresses colorectal cancer liver metastasis, revealing an inflammation-driven immune reprogramming mechanism [71]. In parallel, cabbage-derived EV-like nanoparticles (Cr-ELNs) efficiently encapsulate miR-184 and enter colon cancer cells, demonstrating the operability of oral or local delivery of nucleic-acid therapeutics. Rice-bran nanoparticles (rbNPs) induce cell-cycle arrest and apoptosis, downregulate β-catenin and Cyclin D1, and significantly inhibit tumor progression in a mouse peritoneal dissemination model, providing oncologic support for valorizing an agricultural by-product [72].

In breast cancer, natural vesicles from tea leaves and Camellia flowers have repeatedly been shown to suppress primary tumor growth and lung metastasis. Reported mechanisms include induction of intratumoral reactive oxygen species, mitochondrial damage, and concurrent remodeling of the gut microbiota; oral administration yields sustained antitumor effects in mouse models [59]. Related reviews and databases consistently highlight the plant miR-159–TCF7 axis as a key pathway: synthetic or plant-derived miR-159 downregulates TCF7, inhibits Wnt–MYC-related signaling, and suppresses tumor proliferation in mice while exerting relatively mild effects on normal mammary epithelial cells, indicating tumor selectivity for the combination of nucleic-acid cargo with plant vesicles [73]. Additional in-vitro evidence shows that ginger-derived vesicles induce apoptosis and cell-cycle arrest and inhibit migration in the triple-negative breast cancer line MDA-MB-231, further extending applicability [74].

For melanoma and cancer immunotherapy, recent work shows that ginger-derived EVs are selectively taken up by commensal gut bacteria. Their cargo, aly-miR159a-3p, suppresses phospholipase C expression in recipient bacteria and promotes docosahexaenoic acid accumulation in tumor cells, which subsequently downregulates PD-L1 and significantly enhances the efficacy of anti-PD-L1 therapy—illustrating cross-domain regulation along a “plant vesicle–microbiota–tumor immunity” axis [75]. In brain tumors, grapefruit-derived nanovectors have been shown to cross the brain barriers to deliver miR-17 into intracranial tumors, inhibiting growth and prolonging survival. This demonstrates delivery feasibility in central nervous system malignancies and complements the leukocyte-homing strategy used for inflamed tumors [76]. At present, there are no systematic advances reported for ADEVs in cancer research.

### 5.3. Metabolic Disorders & Hepatobiliary Diseases

In recent years, research on PDEVs in metabolism has progressed from mitigating inflammation and oxidative stress along the gut–liver axis to directly modulating intestinal transporters and the bile-acid cycle at the molecular level. In parallel, animal studies and formulation models have verified oral usability and local on-site efficacy [77]. For non-apple sources, evidence first emerged as organ-level protection [7,78]. By contrast, ADEVs have established a relatively clear mechanistic chain in human intestinal epithelial transporters and bile-acid pathways.

Using ginger-derived nanoparticles (GDNPs) as an example, oral administration in mice significantly alleviates alcohol-induced liver injury, lowers serum and histological damage indices, and improves gut–liver axis inflammation and oxidative stress [78]. Subsequent work indicates that related plant vesicles can activate Nrf2/HO-1 and inhibit NF-κB to ameliorate alcoholic liver injury, providing pathway-level support for applications in metabolic liver disease [79]. More broadly, edible-plant vesicles have been highlighted as potential interventions for metabolic disorders: their lipid membranes together with nucleic acids, proteins, and small molecules can jointly modulate oxidative stress, inflammation, epithelial barrier function, and the gut microbiota, thereby influencing glucose and lipid metabolism and offering a rationale and early models for improving insulin sensitivity and hepatic lipid handling [36]. Additionally, exosome-like extracellular vesicles derived from Pueraria lobata alleviate osteoporosis by enhancing autophagy (preclinical evidence), highlighting the potential of PDEVs in metabolic bone disorders [80].

Consistent with these observations, ADEVs have delineated a coherent mechanism in human intestinal epithelial models that spans cellular uptake, molecular targets, and functional readouts. In Caco-2 cells and heterologous reporter systems, ADEVs uptake significantly reduces OATP2B1 (gene SLCO2B1) mRNA and protein levels and suppresses substrate transport. This inhibition depends on the SLCO2B1 3′UTR and is lost when vesicles are heat- or ultrasound-disrupted, pointing to post-transcriptional regulation mediated by macromolecular cargo carried by the vesicles [7]. Further dissection shows functional binding between several apple miRNAs and the SLCO2B1 3′UTR; among these, mdm-miR-7121d-h has the most direct support: an inhibitor partially reverses suppression, a mimic downregulates endogenous SLCO2B1, and mutating the recognition site abolishes reporter repression, indicating an RNA-induced silencing complex–mediated post-transcriptional mechanism [81].

Beyond drug-uptake transporters, ADEVs indirectly regulate bile-acid reclamation at the transcription-factor level. In Caco-2 cells, ADEVs treatment lowers ASBT expression (gene SLC10A2) at both mRNA and protein levels and reduces bile-acid uptake. Mechanistic studies indicate that this effect does not arise from direct action on the SLC10A2 3′UTR; rather, plant miRNAs delivered by vesicles downregulate RARα (gene NR1B1) expression and stability, weakening its transcriptional activation of the SLC10A2 promoter and ultimately decreasing ASBT expression and function [9]. These findings suggest that ADEVs may influence the enterohepatic circulation of bile acids and thereby modulate cholesterol metabolism and related phenotypes, warranting further in vivo quantification.

### 5.4. Mucosal Inflammation and Barrier Repair (IBD, Colitis, Oral Mucosa)

In gastrointestinal mucosal diseases, the role of PDEVs has expanded from a purely anti-inflammatory effect to an integrated therapeutic framework encompassing barrier reconstruction, immune reprogramming, microbiota modulation, and targeted delivery. In models of colitis and colitis-associated cancer (CAC), oral administration of ginger-derived extracellular vesicles significantly attenuates dextran sulfate sodium (DSS)—and tumor-related inflammation, suppresses pro-inflammatory pathways, and improves histopathology, thereby establishing the feasibility of local intestinal activity after oral PDEVs delivery [82,83]. At the mechanistic level, broccoli-derived nanoparticles activate the AMP-activated protein kinase (AMPK) pathway in dendritic cells and drive a tolerogenic phenotype, which reduces inflammation and protects the mucosa across three murine colitis models. These findings directly link oral PDEVs to the establishment and maintenance of mucosal tolerance via immunometabolic pathways [84]. Consistently, ginseng-derived EV-like vesicles mitigate disease progression in DSS colitis and remodel the intestinal immune microenvironment, further supporting a role for edible plant vesicles in mucosal immune regulation [85].

On the delivery front, grapefruit-derived nanovectors functionalized with activated leukocyte receptors home selectively to inflamed tissues by leveraging a cascade of selectins, chemokines, and integrins. This approach increases lesion accumulation and delivery efficiency across multiple inflammation-driven models and outlines an engineering route for inflammation-site targeting [71]. In addition, reviews and recent primary studies show that EV-like vesicles from citrus and green tea improve epithelial tight junctions, alleviate oxidative stress, and modulate the gut microbiota, providing systematic evidence for the coordinated relationships among barrier homeostasis, the microbiota, and mucosal immunity [54].

Within the same framework of mucosal inflammation and barrier repair, ADEVs have yielded preliminary signals in clinical populations. In client-owned dogs with inflammatory bowel disease, ten days of oral administration led to significant reductions in fecal secretory IgA and calprotectin toward healthy ranges, a shift in the gut microbial community toward a healthy profile, and lower endoscopic and ultrasonographic disease-activity scores—findings consistent with symptom improvement via modulation of mucosal immunity and the microbiota [86]. Concordant in vitro data show that apple extracellular vesicles are taken up by immune and epithelial cells, downregulate IL-1β and IL-8, and are enriched for an anti-inflammatory miRNA network exemplified by miR-146a, supporting a molecular basis for immunomodulation within the mucosal microenvironment [8]. Pharmacological studies further indicate that ADEVs enter Caco-2 and LS180 cells via clathrin-mediated endocytosis and are detectable at the basolateral side of murine intestinal villi, demonstrating the feasibility of local exposure and trans-epithelial delivery after oral dosing and informing formulation development [9]. Given their lipid composition and gastrointestinal stability, PDEVs can traverse the gastric barrier and exert distant-organ effects after oral administration; therefore, combining local (e.g., topical or site-directed) application with oral delivery may offer synergistic benefits in future therapeutic designs. With respect to the oral mucosa, clinical evidence remains at an early stage; randomized, controlled studies with objective, quantitative endpoints are needed for further validation.

### 5.5. Immune and Vascular Systemic Diseases (IgA Nephropathy and Vascular Calcification/Atherosclerosis)

In the context of systemic immune and vascular diseases, research on PDEVs is shifting from broad anti-inflammatory and antioxidant actions toward strategies that achieve microenvironment-guided targeting while reprogramming immune-cell phenotypes and endothelial responses.

For immune-mediated kidney disease represented by IgA nephropathy, the most translationally oriented evidence comes from an oral regimen in which orange-derived extracellular vesicles are loaded with dexamethasone phosphate (EVs–DexP). In a murine IgA nephropathy model, oral EVs–DexP reduced proteinuria more effectively than the free drug and mitigated glomerular and tubular pathology. These effects coincided with uptake of vesicles by submucosal lymphocytes in the ileocecal region, reduced IgA^+^B220^+^ cells in Peyer’s patches, and decreased gut-derived IgA production, suggesting that mucosal immune reprogramming along the gut–kidney axis can drive systemic anti-inflammation and renal benefit [87,88].This study provides a prototype of oral, site-directed immunosuppression using edible-source vesicles combined with a glucocorticoid, supported by both disease endpoints and immunologic readouts.

Current vascular evidence converges on two hubs—endothelial inflammation and macrophage phenotype. Lemon-derived EV-like vesicles suppress ERK and NF-κB signaling and activate AhR and Nrf2 pathways in vitro and in vivo, thereby lowering pro-inflammatory cytokines and oxidative-stress readouts; such bidirectional modulation of endothelial and immune cells may alleviate endothelial dysfunction, an early driver of atherogenesis [89]. Along a more engineering-oriented route, grapefruit-derived nanovectors that mimic the leukocyte adhesion cascade achieve preferential homing to inflamed sites—sequentially leveraging selectins, chemokines, and integrins—thereby increasing tissue retention and delivery efficiency across inflammation-driven models and offering a transferable strategy for site-specific therapy in inflamed atherosclerotic plaques [90]. In parallel, reviews highlight that extracellular vesicles participate in amplifying inflammation and disturbing lipid metabolism across multiple stages and cell types in atherosclerosis (endothelial, smooth-muscle, macrophage, foam cells). By contrast, vesicles from edible plants, owing to low immunogenicity and oral accessibility, are viewed as safer anti-inflammatory and antioxidant delivery chassis suited for precision interventions in vascular disease [91]. Pilot data further suggest that mulberry (Mori fructus) vesicles can modulate lipid profiles via miRNA combinations and slow atherosclerosis progression, pointing to a “food-derived vesicle—lipid metabolism—arterial disease” axis that merits multi-center validation and pharmacokinetic refinement [92].

In summary, for IgA nephropathy there is animal-level evidence for an oral strategy that combines edible vesicles with an immunotherapeutic agent, with concordant efficacy endpoints and mucosal-immunity mechanisms [87]. For vascular disease, the focus centers on endothelial and macrophage pathways: on one side, intrinsic anti-inflammatory and antioxidant activity of the material (exemplified by lemon-derived vesicles [89]), on the other, engineering of inflammation-site targeting (exemplified by grapefruit-derived vectors [76],complemented by miRNA-based metabolic modulation signals such as those from mulberry vesicles [92]) Notably, to date we have not identified animal efficacy or clinical-translation reports for ADEVs in IgA nephropathy, atherosclerosis, or vascular calcification.

### 5.6. Neurological Disorders

In the neurological field, research on PDEVs is moving beyond generic anti-inflammatory and antioxidant effects toward mechanism-driven studies centered on central nervous system (CNS) delivery routes, coordination between microglia and peripheral immunity, and molecular targets such as mitochondria and the NLRP3 inflammasome. Correspondingly, CNS-oriented pharmaceutic paradigms are emerging, including intranasal administration, biomimetic surface functionalization, and oral delivery that engages the gut–brain axis to modulate neuroinflammation [93]. Reviews note the low immunogenicity and favorable biocompatibility of PDEVs [37] and their potential to achieve CNS exposure by crossing the blood–brain barrier or via the nose-to-brain pathway, offering new carrier options for neurodegenerative and intracerebral inflammatory disorders [76].

Grapefruit-derived nanovectors modified with folate and hybridized with polyethylenimine have delivered small nucleic acids to the murine brain parenchyma via the intranasal route, with uptake by target cells. Although demonstrated in a brain-tumor model, this establishes the feasibility of brain entry and delivery and provides a methodological reference for non-oncologic CNS diseases [76]. Ginger-derived EV-like nanoparticles suppress the NLRP3 inflammasome and reduce pro-inflammatory cytokines, suggesting potential to alleviate neuroinflammation and protect neurons [94]. Vesicles from Atractylodes rhizome similarly inhibit lipopolysaccharide-induced activation of microglia, supporting the reproducibility of “PDEVs-mediated microglial phenotype modulation” [95]. In addition, reviews and primary studies have proposed that orally administered ginger vesicles may indirectly attenuate brain inflammation through gut–brain communication, highlighting the gut–brain axis as an auxiliary route worthy of further quantitative validation [96].

Despite accumulating evidence that PDEVs carry diverse small RNAs and other nucleic acids, the concept of bona fide cross-kingdom regulation remains controversial. While several reports have described uptake of plant miRNAs by mammalian cells and downstream gene modulation, other studies have failed to reproduce these findings under stringent controls, raising concerns about contamination, technical artefacts, or differences in isolation workflows. To address these uncertainties, we recommend that future ADEVs studies implement rigorous experimental controls—such as RNase treatment (with and without detergent), the use of exosome-depleted media, spike-in synthetic RNA controls, sham (heat- or sonication-disrupted) vesicle controls, and parallel negative controls for cross-species contamination—together with quantitative tracking and standardized sequencing pipelines. Establishing such methodological minimal standards will be essential for resolving discrepancies in the literature and for validating the biological relevance of nucleic-acid cargo in translational applications.

## 6. Conclusions and Outlook

Research on PDEVs is advancing from “concept and in vitro activity” toward “engineering-ready delivery and validation in animal models.” Within this landscape, ADEVs have generated a relatively continuous body of evidence across isolation and characterization, regulation of epithelial transporters and bile-acid pathways, mucosal immunity, and skin-barrier biology, along with early signals for usability and safety in human settings. However, compared with systems such as ginger, grapefruit, and tea, ADEVs still lack systematic animal efficacy and translational data in high-burden indications, including cancer and vascular disease.

Future ADEVs studies must therefore prioritize process standardization and the harmonization of critical quality attributes (CQAs). Establishing this foundation requires not only multi-omics profiling to establish reproducible dose–response relationships, but also immediate focus on the major hurdles to clinical translation that demand pharmaceutical-grade control.

### 6.1. Regulatory Landscape and Quality Standards

The regulatory path for plant-derived nanocarriers remains ambiguous, potentially classifying ADEVs as New Dietary Ingredients (NDI), Generally Recognized as Safe (GRAS) substances (in the US), or eventually as a Drug/Biological Product. Clarification is essential, as the final regulatory designation dictates the required manufacturing standard (e.g., cGMP) and the stringency of preclinical safety packages. Establishing harmonized CQAs (e.g., purity, particle identity, and in vitro potency) is a prerequisite for any regulatory submission.

### 6.2. Industrial Scalability and Process Selection

Direct extraction from edible plant tissue (e.g., apple pulp) is the most suitable approach for achieving industrial scalability and is preferred over methods like plant cell-culture derivation. Cell-culture systems present significant challenges in eliminating residual growth factors and antibiotics, which compromise safety and purity. Scalability must be built upon robust and reproducible isolation methods (e.g., tangential flow filtration) to maintain vesicle integrity and yield when processing large volumes of raw material.

### 6.3. Agricultural Variability, Raw Material Control, and Contamination

The biological origin of ADEVs introduces significant agricultural variability (cultivar, location, growing conditions). Crucially, since PDEVs act as scavenging carriers, raw materials sourced from intensive farming may lead to the bio-concentration and enrichment of pesticides and microbicides, thus posing a safety hazard. Furthermore, suboptimal growing conditions reduce PDEVs yield and bioactivity. Future research must establish stringent Raw Material Quality Control (RMQC) standards, including mandatory, validated testing for pesticide and heavy metal residues, to ensure source purity and final product quality consistency.

In parallel, oral, transdermal, and intranasal delivery should be mapped with in vivo biodistribution, barrier transit, and clearance kinetics while filling gaps in repeat-dosing and long-term toxicology data for systemic exposure. Finally, research should concentrate on mucosal and cutaneous contexts where mechanistic rationales already exist, closing the loop from mechanism to animal studies to human evidence. In addition, ADEVs, by virtue of their low immunogenicity, should be leveraged as carriers for approved small molecules or nucleic acids to seek synergy in metabolic and tumor-microenvironment models. Collectively, these efforts will help drive the transformation of sustainable agri-food resources into safe, low-cost, scalable bionanocarriers, opening new intervention avenues at the interface of nutrition and pharmacology.

## Data Availability

No new data were created or analyzed in this study. Data sharing is not applicable to this article.
